# Learning Moiré Pattern Elimination in Both Frequency and Spatial Domains for Image Demoiréing

**DOI:** 10.3390/s22218322

**Published:** 2022-10-30

**Authors:** Chenming Liu, Yongbin Wang, Nenghuan Zhang, Ruipeng Gang, Sai Ma

**Affiliations:** 1 State Key Laboratory of Media Convergence and Communication, Communication University of China, Beijing 100024, China; 2 Academy of Broadcasting Science, National Radio of Television Administration, Beijing 100866, China; 3 Key Laboratory of Convergent Media and Intelligent Technology, Ministry of Education, Communication University of China, Beijing 100024, China

**Keywords:** moiré patterns, image demoiré, frequency domain, wavelet transform

## Abstract

Recently, with the rapid development of mobile sensing technology, capturing scene information by mobile sensing devices in the form of images or videos has become a prevalent recording method. However, the moiré pattern phenomenon may occur when the scene contains digital screens or regular strips, which greatly degrade the visual performance and image quality. In this paper, considering the complexity and diversity of moiré patterns, we propose a novel end-to-end image demoiré method, which can learn moiré pattern elimination in both the frequency and spatial domains. To be specific, in the frequency domain, considering the signal energy of moiré pattern is widely distributed in the frequency, we introduce a wavelet transform to decompose the multi-scale image features, which can help the model identify the moiré features more precisely to suppress them effectively. On the other hand, we also design a spatial domain demoiré block (SDDB). The SDDB module can extract moiré features from the mixed features, then subtract them to obtain clean image features. The combination of the frequency domain and the spatial domain enhances the model’s ability in terms of moiré feature recognition and elimination. Finally, extensive experiments demonstrate the superior performance of our proposed method to other state-of-the-art methods. The Grad-CAM results in our ablation study fully indicate the effectiveness of the two proposed blocks in our method.

## 1. Introduction

In recent years, mobile phones have greatly changed our lives and are widely used in many scenes [1,2]. They can help us to record important information in time by shooting images or videos. However, when we use mobile phones to record scenes with LED screens or display screens, the images or videos tend to have wavy interference patterns, known as moiré patterns. The reason for this is that when the photosensitive elements in the scanning instrument and digital camera are disturbed by high frequency, two equal amplitude sine waves with similar frequencies are superimposed, and the amplitude of the synthesized signal will change according to the difference of the two frequencies; then, colored and irregular-shaped stripes will appear on the images [3]. One way to avoid moiré patterns is to improve the camera resolution, making it larger than the resolution of the captured screen, but this is very expensive. Another way is to add a low-pass filter in front of the sensing device of the camera to reduce the moiré patterns, but the disadvantage of this is that the addition of the low-pass filter will impair the image details, making the image content blur.

With the rapid development of deep learning, using algorithms based on deep learning for image demoiréing has received considerable attention. For example, Liu et al. [4] considered that the energy distribution of moiré patterns is relatively concentrated in the frequency domain and used discrete cosine transform (DCT) to transfer the image from the spatial domain to the frequency domain. They decomposed the image into texture components and moiré components in the frequency domain so as to better eliminate the high-frequency moiré patterns in the images. However, it is difficult to completely eliminate moiré patterns only through the frequency domain. Yang et al. [5] used layer decomposition on polyphase components (LDPC) to decompose the image into a background layer and a moiré layer. In addition, to better remove the moiré patterns, the method was applied on Y and RGB channels. Sun et al. [6] proposed a multi-resolution network (DMCNN) for the multi-frequency characteristics of moiré patterns. However, this network cannot deal with low frequencies and large color blocks. Some images have poor visual performance. In 2020, He et al. [7] proposed a full high-definition demoiréing network (FHDe${}^{2}$Net) to solve the problem of high-resolution image demoiréing. They used a global-to-local pattern removal strategy for fine detail preservation in high-resolution images and adopted DCT to transform the image into the frequency domain so as to better address the problems of the wider moiré pattern scale range. In 2022, Yu et al. [8] proposed ESDNet, a method for demoiréing in 4K ultra-high definition. In order to eliminate the multi-scale moiré patterns, they built a semantic-aligned scale-aware module. However, most existing methods attempted to eliminate the moiré patterns only in the frequency domain or the spatial domain. Moiré patterns have a certain diversity and complexity. In the frequency domain, moiré patterns span low frequencies and high frequencies. In the spatial domain, moiré patterns mix with the image texture seriously and also cause color distortions. It is difficult to completely remove moiré patterns while keeping the original texture just from one domain.

To address the above-mentioned problems, we propose a novel and effective end-to-end demoiré network, which can eliminate the moiré patterns both in frequency and spatial domains, named FSD-Net. Specifically, in the frequency domain, we introduce the wavelet transform to decompose the multi-scale image features, which can help the network better identify the moiré features so as to suppress the moiré features in the image generation. In the spatial domain, we design a spatial domain demoiré block (SDDB), which can extract the moiré features from the mixed image features. After the extraction, the moiré features will be subtracted from the mixed image feature, which can obtain clean features to generate a clean image without moiré patterns. By demoiréing both in the frequency domain and in spatial domain, our proposed network can obtain superior performance regarding eliminating moiré patterns. Experimental results demonstrate that our proposed method outperforms several state-of-the-art demoiré methods.

The contributions of our research can be summarized as follows:

- We propose a novel method to eliminate moiré patterns both in the frequency domain and spatial domain. Experimental results indicate that our method achieves state-of-the-art performance compared with other methods.
- We introduce wavelet transform to decompose the multi-scale image features, Which may help the network to better identify the moiré features so as to suppress the moiré features during the image generation.
- We design a spatial-domain demoiré block, which can effectively extract moiré features from mixed image features. Then we can subtract the moiré features from the mixed features to obtain clean features, which are used during the image generation.

## 2. Related Work

### 2.1. Traditional Methods

Moiré patterns are often caused by the aliasing of two equal-amplitude sine waves with similar frequencies. The direct solution is to change the frequency of one sine wave. In 2000, Nishioka et al. [9] proposed that adding a low-pass filter in front of the digital camera lens could effectively remove the moiré patterns. This method can avoid the moiré patterns during the shooting, though it also loses some details and texture information. Another method is to upgrade the camera with a higher resolution photosensitive element, but such a camera is expensive and cannot fundamentally solve the moiré problem. Sidorov et al. [10] proposed a spectral model, which leverages the magnitude of the Fourier spectrum of the image to identify the moiré patterns. In addition, signal decomposition is also an important method for removing moiré patterns. Yang et al. [11] proposed a novel image demoiréing method by signal decomposition and guided filtering. Firstly, they adopted a low-rank and sparse matrix decomposition model to remove moiré patterns in the green (G) channel. Then, they removed moiré patterns in red (R) and blue (B) channels via guided filtering by the obtained texture layer of the G channel.

### 2.2. Deep Learning Methods

Unlike with other image restoration tasks, such as image denoising [12,13,14], image dehazing [15,16], and image demosaicing [17,18], the difficulty of image demoiréing is how to remove moiré patterns with various frequencies and color distortion. With the widespread popularity of deep learning, deep convolutional neural networks have also been applied to image demoiréing. In 2018, Sun et al. [6] first proposed a multi-resolution fully convolutional neural network DMCNN for multi-frequency moiré feature elimination. In 2019, Gao et al. [19] considered that the relationship among multi-scale features is significantly ignored and designed a feature-enhancing branch to fuse high-level features with low-level ones, which can restore the image details during image demoiréing. In 2019, Cheng et al. [20] also dealt with multi-scale features and proposed a dynamic feature-encoding module, which can encode the variations of moiré patterns. He et al. [21] proposed a Moiré pattern removal neural network (MopNet) based on DenseNet. The model integrated the moiré frequency distribution, edge intensities, and appearance categories into design learning modules. However, when irregular and unstable backgrounds such as sand and stone ground are encountered, the edge features of the pattern are difficult to determine and need to be improved. In 2020, Zheng et al. [22] proposed a novel multi-scale bandpass convolutional neural network (MBCNN), which splits image demoiréing into two steps: moiré texture removal and tone mapping. In 2020, Liu et al. [23] proposed a WDNet network, including a direction perception module. The module can carry out the convolution operation in 8 different directions so as to better capture the spatial distributions of moiré patterns. He et al. [7] proposed a full high-definition demoiréing network (FHDe${}^{2}$Net) to solve the high-resolution image demoiréing by a cascade of two networks focused on global and local level moiré removal, respectively. Moreover, they also proposed a lower high-resolution content separation branch, which can preserve the fine details against the distortions in demoiré processing. In 2021, Park et al. [24] proposed an unsupervised end-to-end moiré pattern removal method based on cyclic moiré learning. Compared with other methods, this method used an unpaired set of clean and moiré images. In 2022, Yu et al. [8] proposed a baseline model, ESDNet, a method for removing moiré patterns in 4K ultra-high-definition images, and constructed a semantic-aligned scale-aware module to solve the moiré elimination effectively. In the same year, Dai et al. [25] proposed the first moiré pattern removal method with implicit feature space alignment and selective feature aggregation for hand-held video.

### 2.3. Wavelet-Based Methods

The wavelet transform can decompose complex and composite information into elementary simple forms at different positions and scales, which can benefit us in understanding the information. Wavelet-based methods have been explored in several computer vision tasks. For example, Lotfi et al. [26] adopted the Daubechies 4 wavelet transform and first-order color moments to represent the image information, then a neural network was proposed to identify the category of aircraft images. Nayak et al. [27] utilized a two-dimensional discrete wavelet transform for extracting brain magnetic resonance image features. Liu et al. [28] used wavelet-based methods to capture age-related texture details at multiple scales in the frequency domain. Huang et al. [29] used wavelets for face super-resolution, where neural networks were used to predict the wavelet coefficients.

## 3. Methodology

In this section, we first describe the structure of our proposed FSD-Net for image demoiréing and the overall pipeline. Next, we present the details of the frequency domain demoiré block and the spatial domain demoiré block, which are the crucial components of FSD-Net. After that, we present the loss function.

### 3.1. Overall Network

**Figure 1:**
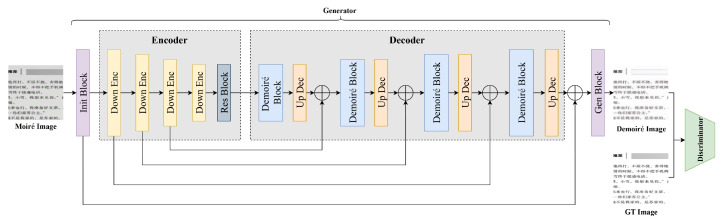
The overall structure of our proposed FSD-Net, which consists of a generator and a discriminator. The generator is designed following the image encode-decode structure.

The overall structure of our proposed FSD-Net is shown in Figure 1. As can be seen, our network consists of a generator and a discriminator. The generator is a U-shaped network with skip connections between the encoder and the decoder. In the encoder, we adopt four down encoder Blocks and a res block to encode the multi-scale moiré image features. Using the decoder, we gradually remove the moiré patterns from the multi-scale feature maps both in the frequency domain and spatial domain by our proposed demoiré block. The discriminator is designed following the structure of [30], which can help further enhance the quality of the generated images.

To be specific, the input of FSD-Net is a moiré image with the size of $I\in R^{3\times H\times W}$. Firstly, we adopt an init block to obtain the initial image feature maps, denoted as $f_{e}^{0}\in R^{C\times H\times W}$, where *C*, *H*, and *W* are the channel, height and weight size of the feature maps, respectively. Then, the initial feature maps will be sent into the encoder, which consists of four down encoder blocks and a res block. Each down encoder block consists of a 3×3 convolution with stride 2, an instance normalization, and a ReLU function. The res block is used to further encode the image feature maps; the output of the encoder is $f_{e}^{4}\in R^{16C\times (H/16)\times (W/16)}$.

Next, following the encoder of the generator, the feature maps $f_{e}^{4}$ are passed through four stages of decoding. Each stage contains a stack of the proposed demoiré block and an up dec block. The motivation of the demoiré block is related to the two major challenges during moiré pattern elimination. One is that the moiré patterns exhibit considerable variation in frequency. The other is that in the spatial domain, the moiré features are seriously mixed with image texture. It is difficult to filter out the moiré patterns under these problems. To address the above-mentioned issues, we built the demoiré block with two core designs: the frequency domain demoiré block (FDDB) and the spatial domain demoiré block (SDDB). In the demoiré block, we first put the decoder feature maps $f_{d}^{i}$ into the FDDB, which can recognize the moiré feature maps in the frequency domain by wavelet transform and then weight the moiré feature maps to suppress them during the image generation. Then, the SDDB is added at the end of FDDB to further remove the moiré features, which are mixed with image texture features by subtracting operations, to obtain cleaner features. After the demoiré block, we adopt a transposed convolution operation in the up-decoder block to decode the image features. Finally, we adopt a gen block to restore the image to its original image size.

### 3.2. Frequency Domain Demoiré Block

The key innovation of our proposed FDDB is to disentangle image decoding feature maps into multiple frequency sub-bands and recognize the moiré features in the frequency domain, then suppress the moiré features by weighting coefficients. We introduce wavelet transform (WT) to transform image features from the spatial domain to the frequency domain. Wavelet transform consists of a low-pass filter and a high-pass filter; it applies the low-pass filter and high-pass filter alternately along feature columns and rows to produce four sub-band frequency features, denoted as LL, LH, HL, and HH, where L indicates the low frequencies and H represents the high frequencies. By using the wavelet transform, the model can better distinguish the moiré features and image features.

**Figure 2:**
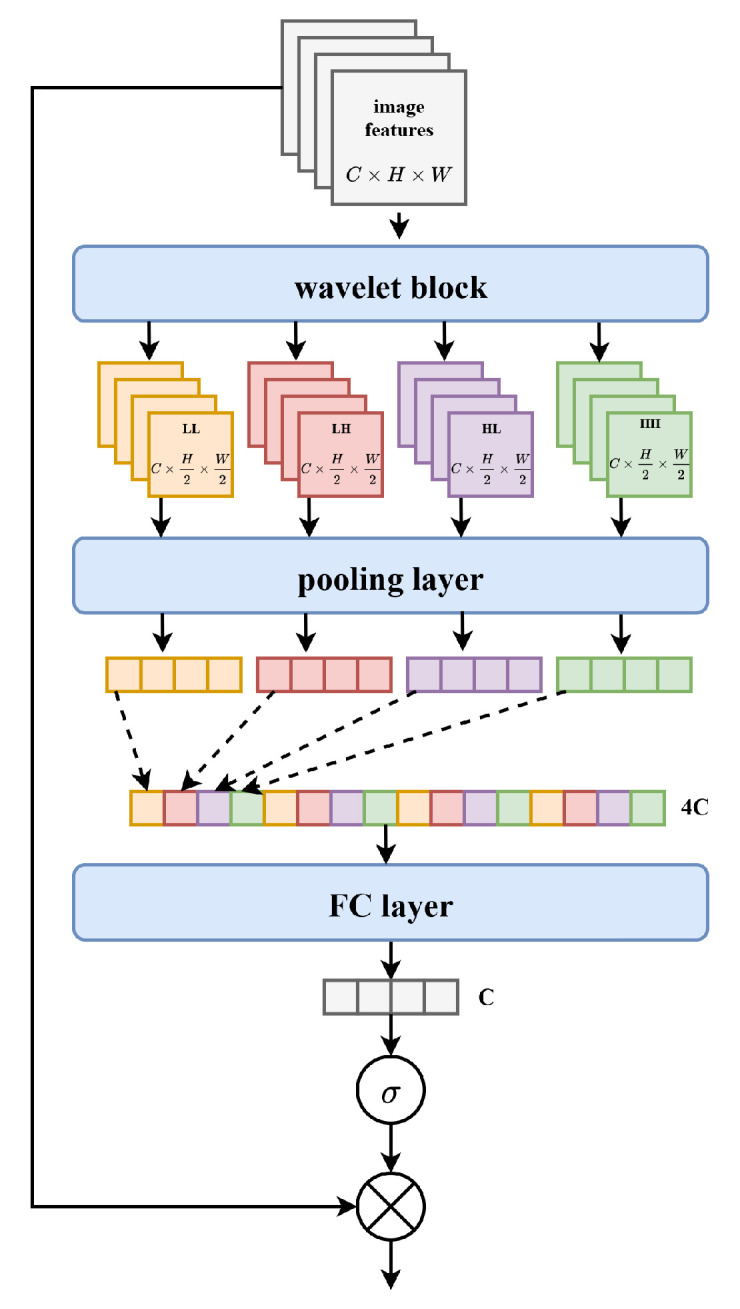
The architecture of the frequency domain demoiré block (FDDB).

The structure of the frequency domain demoiré block is shown in Figure 2. As shown in the figure, we first adopt wavelet transform to decompose features into multiple wavelet sub-bands with different frequency contents. The operation is defined as:

(1) $$f_{d}^{LL},f_{d}^{LH},f_{d}^{HL},f_{d}^{HH}=WT\left(f_{d}\right),$$

where $f_{d}\in R^{C\times H\times W}$ is the input image features, $f_{d}^{LL},f_{d}^{LH},f_{d}^{HL},$ and $f_{d}^{HH}$ represent the four sub-bands, the size of the sub-band feature map is C×H/2×W/2, and WT() denotes the wavelet transform function.

Once the four sub-bands are obtained, we adopt a pooling layer to obtain the feature vector. In this step, it is important to align the low frequency and high frequency pooling parameters of the same input feature map. So after the pooling layer, the four sub frequency domain pooling parameters of each feature map is arranged with the corresponding positions.

Finally, the flattened vector will be sent to a FC layer and a sigmoid function to obtain the weighted parameters, which will be used to weight the input image features so as to suppress the moiré features and obtain clean image features.

### 3.3. Spatial Domain Demoiré Block

**Figure 3:**
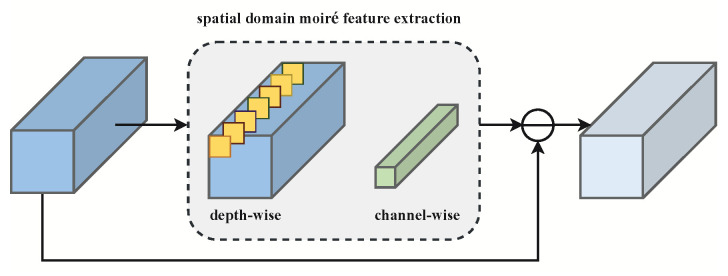
The structure of the spatial domain demoiré block (SDDB).

The above FDDB based on wavelet transform focuses on the recognition of frequency differences between the feature maps. However, some moiré patterns are mixed with the original image texture, and it is difficult to discriminate one from the other; the FDDB suffers from a limited capability to remove the moiré patterns in this case. To address this limitation, we design a structure that removes moiré patterns directly in the spatial domain. We achieve this by designing a spatial domain demoiré block, which is illustrated in Figure 3. In the SDDB, we leverage the depth-wise convolution operation to extract the moiré features from the mixed features. Then, we use a channel-wise convolution operation to obtain refined moiré features. Finally, we obtain the clean image features by subtracting the moiré features from the original input features. These operations can be defined as follows:

(2) $$f_{m}=Conv\left({\hat{f}}_{d}\right),$$

(3) $${\hat{f}}_{d}^{*}={\hat{f}}_{d}⊖f_{m},$$

where Conv() denotes both the depth-wise convolution and the channel-wise convolution, and ⊖ denotes the feature-wise subtraction operation.

### 3.4. Loss Function

In our research, to obtain better demoiréing performance, we adopt a combined loss function, including content loss $L_{ct}$, perceptual loss $L_{per}$, and adversarial loss $L_{adv}$. The overall loss function of the generator can be formulated as:

(4) $$L=\lambda_{ct}\times L_{ct}+\lambda_{per}\times L_{per}+\lambda_{adv}\times L_{adv}^{G},$$

where $\lambda_{ct}$, $\lambda_{per}$, and $\lambda_{adv}$ are the balancing parameters of content loss, perceptual loss, and adversarial loss, respectively.

**Content Loss.** We use the L1 loss to measure the content loss between the GT image and the demoiréing image generated by our proposed network.

(5) $$L_{ct}={∥I_{gt}-I_{de}∥}_{1},$$

where $I_{gt}$ denotes the GT image, and $I_{de}$ denotes the demoiréing image.

**Perceptual Loss.** To penalize the perceptual and semantic discrepancy, we adopt the pre-trained VGG-19 network to extract the features of the GT image $I_{gt}$ and the features of the demoiréing image $I_{de}$. Then, we use the L1 loss to measure the perceptual loss. The formula is defined as follows:

(6) $$L_{per}=\sum_{i}(∥\phi_{i}\left(I_{gt}\right)-\phi_{i}\left(I_{de}\right){∥}_{1}),$$

where ϕ() denotes the VGG-19 network and *i* denotes the *i*-th layer of VGG-19 network. In our experiments, we employ the feature maps of the four layers conv2_2, conv3_4, conv4_4, and conv5_4 to calculate the perceptual loss.

**Adversarial Loss.** To effectively synthesize a realistic image, we introduce an adversarial loss, which can promote the generator to create a realistic demoiré image. We use the binary cross-entropy criterion with a softmax function to calculate the loss value. The loss function for the generator is defined as:

(7) $$L_{adv}^{G}=-log\left(D\left(I_{de}\right)\right);$$

and the loss function for training the discriminator is defined as:

(8) $$L_{adv}^{D}=-log\left(D\left(I_{gt}\right)\right)-log\left(1-D\left(I_{de}\right)\right);$$

where D() represents the discriminator.

## 4. Experiments

In this section, we first describe the dataset used in our experiment and the implementation details. Then, we report the subjective and objective evaluation results in comparison with other state-of-the-art methods to demonstrate the effectiveness of our proposed demoiré method. Finally, we conduct some ablation studies to verify the performance benefit brought by each functional component in our method.

### 4.1. Dataset and Implementation Details

**Dataset.** Our experiments are conducted on the dataset provided by the Document Image Demoiré Contest, which is a sub-competition of the Baidu NetDisk AI competition. All images in the dataset are collected from real-world scenes. The dataset consists of 1000 training samples and 200 test samples; each sample contains an image with moiré patterns and a ground-truth image without moiré patterns.

**Implementation Details.** The discriminator in our proposed method has a similar architecture to [30], which has an input size of 256×256. To achieve a good discriminative performance and accommodate the input size of 512×512 in our paper, we increased the depth of the discriminator network. We set the initial learning rate to $2\times 10^{-4}$ and $1\times 10^{-4}$ for the generator and the discriminator, respectively. Then, the two learning rates were both decayed by 0.1 in the 20th epoch and 40th epoch, respectively. The total training epoch was set to 60. The batch size was set to 10. We adopted the Adam optimizer [31] with $\beta_{1}=0.5$, $\beta_{2}=0.99$ to optimize our generator and discriminator. All input images were resized into 512×512 pixels, and random horizontal flipping was adopted for data enhancement. The balancing parameters $\lambda_{ct},\lambda_{per},\lambda_{adv}$ in the loss function were set to 2.0,1.0, and 1.0, respectively. Our proposed network was implemented with a PyTorch framework and trained with two NVIDIA RTX3090 GPUs.

**Evaluation Metrics.** Following the previous works, we used common metrics to evaluate the demoiré performance: PSNR (peak signal-to-noise ratio) and SSIM (structural similarity). In addition, we also showed the visual comparison with other state-of-the-art methods to evaluate the effectiveness of our proposed method.

### 4.2. Comparison to Other Methods

We compared our method with several state-of-the-art methods, including U-Net [32], WDNet [23], MBCNN [22], FHDe${}^{2}$Net [7], and HRDN [33].

**U-Net** is a very excellent network and is widely used in many image generation and restoration tasks. We chose this model as one of the comparative methods and trained it from scratch.

**WDNet** is a demoiré method based on wavelet transform. In contrast to our proposed method, WDNet first employs 2D fast wavelet transform to decompose the input RGB image into a sequence of wavelet subbands; these subbands will then be sent to the network to remove moiré patterns. Finally, an inverse wavelet transform is adopted to obtain the final demoiré RGB image. Thus, WDNet works mainly in the wavelet domain.

**MBCNN** is a demoiré method based on multi-scale features; it removes the moiré patterns in the frequency domain by the discrete cosine transform (DCT).

**FHDe${}^{2}$Net** is also a method that removes the moiré patterns in the frequency domain by the DCT. This method adopts a cascaded global-to-local moiré pattern removal strategy, which can handle higher-resolution images.

V is a novel high-resolution demoiré network. It also removes the moiré patterns based on frequency domain and multi-scale features. It also fully takes advantage of the relationship among feature maps with different resolutions to exchange information and enhance details.

**The qualitative results.** In order to verify the effectiveness of our proposed method, we report the qualitative results compared with other methods. Table 1 illustrates the qualitative results. As can be seen, our method outperforms them both in PSNR and SSIM, achieving state-of-the-art performance. The results fully demonstrate the effectiveness of our proposed method.

**Figure 4:**
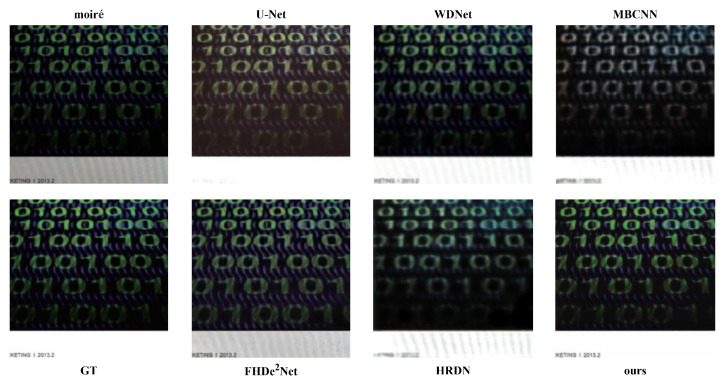
Visual comparisons between our proposed FSD-Net and other methods.

**Figure 5:**
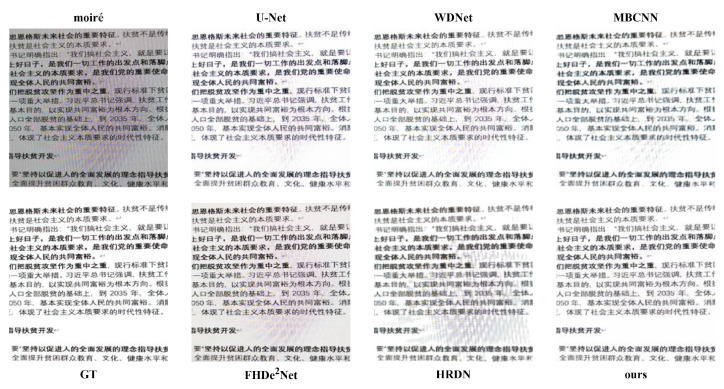
Visual comparisons between our proposed FSD-Net and other methods. The example image contains serious moiré patterns.

**The visual results. **Figure 4 and Figure 5 show visual comparisons with different degrees of moiré patterns. Figure 4 shows the visual results with relatively slight moiré patterns. We note that WDNet and HRDN are unable to totally remove the moiré patterns compared to other methods. U-Net and MBCNN cannot generate clear image content during the demoiréing process. FHDe${}^{2}$Net cannot competently handle the color distortion. In contrast, our method can remove moiré patterns while preserving image details.

Figure 5 shows the visual results with serious moiré patterns. It can be seen that MBCNN cannot reconstruct text details, resulting in image blur. FHDe${}^{2}$Net and HRDN cannot perfectly remove moiré patterns. U-Net and WDNet also cannot obtain an ideal demoiréing performance. Our proposed FSD-Net greatly outperforms the above-mentioned methods and generates a superior visual performance. The visual comparisons further confirm the effectiveness of our method in terms of preserving high-quality details while eliminating moiré patterns.

### 4.3. Ablation Study

To validate the effectiveness of the proposed frequency domain demoiré block and spatial domain demoiré block, we conducted an ablation study, which contains the following variants. (1) Baseline: we adopted only the generator without the frequency domain demoiré block and the spatial domain demoiré block. (2) Baseline+FDB: we added the frequency domain demoiré block on the baseline network. (3) Baseline+SDB: we added the spatial domain demoiré block on the baseline network. (4) Baseline+FDB+SDB: we added both the frequency domain demoiré block and the spatial domain demoiré block to the baseline network. We report the quantitative results in Table 2.

From Table 2, it can be seen that: (1) compared to the baseline, the Baseline+FDB and Baseline+SDB both clearly improve the PSNR and SSIM scores, demonstrating that the two functional blocks both contribute to a performance gain. (2) The combination of the two functional blocks remarkably improves the metrics, further proving the demoiréing solution in both the frequency domain and spatial domain is correct and feasible.

In addition, we further provide the visualization results to better illustrate the effectiveness of our proposed frequency domain demoiré block and spatial domain demoiré block.

**Figure 6:**
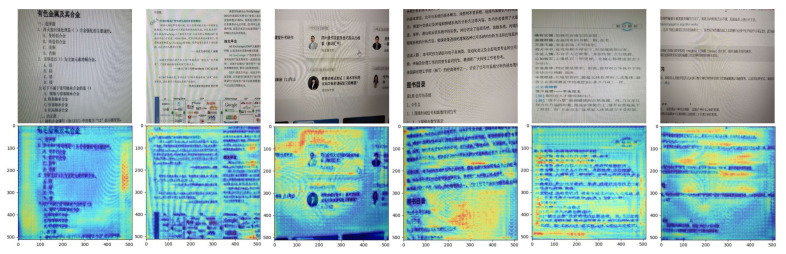
The total visualization results of the four spatial domain demoiré blocks. The first row is the original moiré images. The second row is the Grad-CAM results.

Figure 6 shows the Grad-CAM results generated by the 3×3 convolutional operation within the spatial domain demoiré block. It can be seen that the active areas are the salient moiré regions, not the image content areas. It indicates that our proposed spatial domain demoiré block can focus on capturing the moiré features and suppress the moiré features during the image generation to obtain a clean image.

**Figure 7:**
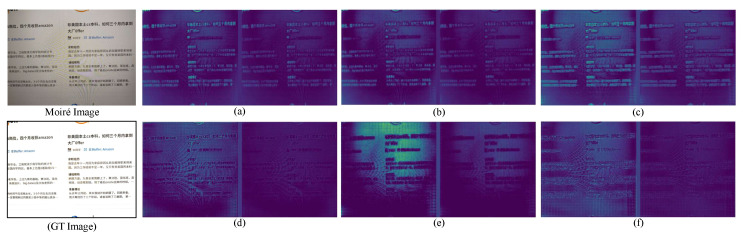
The visualization results of the last frequency domain demoiré block. In (**a**–**f**), the left side is the original feature maps, and the right side is the weighted feature maps by the last frequency domain demoiré block.

To verify the impact of our proposed frequency domain demoiré block, we show the comparison results between the original feature maps and the weighted feature maps by the frequency domain demoiré block. The visualization results are shown in Figure 7. We can see that: (1) in the first row, the left side of (a), (b), and (c) mainly activate the image content, while the corresponding weighted suppression effects in the right side of (a), (b), and (c) are not obvious; (2) In the second row, the features of (d) and (e) mainly represent the moiré regions, so that the weighted suppression is relatively significant. The feature of (f) contains the image content and the moiré content. Our proposed frequency domain demoiré block can effectively identify and suppress the moiré feature. Such visual results prove that our proposed frequency domain demoiré block can assist the model in recognizing and suppressing moiré features.

## 5. Discussion and Conclusions

In this paper, we study how to eliminate moiré patterns more effectively. We summarize and find that previous methods mainly remove moiré patterns in one domain. However, moiré patterns cover a wide range in frequency and will appear in any area with different colors and shapes, making it difficult to eliminate moiré patterns from one domain. To this end, we explore to eliminate moiré patterns both in frequency and spatial domains. In the frequency domain, we introduce wavelet transform to help identify moiré pattern features so as to suppress them. In the spatial domain, we design an SDDB module to remove the moiré features that are mixed with image features. The comparable experiments show that our proposed method is better than other state-of-the-art methods in moiré pattern elimination.

However, there are still several limitations of our demoiré method. First, the reconstruction quality of the demoiré image still has a gap with the ground-truth image, especially in the regions where moiré patterns and image content are seriously mixed. Therefore, the research of content enhancement is our future work, including image deblurring and super-resolution. In addition, we note that our method cannot well preserve the background color if it is similar with some moiré colors. Thus, how to further improve the ability of our method to distinguish moiré features from image features still needs exploration.

## Figures and Tables

**Table 1:** The comparison of qualitative results between our proposed FSD-Net and other methods.

Network	PSNR (dB)	SSIM
U-Net	27.62	0.8289
WDNet	28.66	0.8745
MBCNN	26.83	0.8113
FHDe${}^{2}$Net	26.39	0.8786
HRDN	27.38	0.8626
ours	33.24	0.8970

**Table 2:** Quantitative results of different variants of FSD-Net.

Network	PSNR (dB)	SSIM
Baseline	32.01	0.8717
Baseline+FDB	32.62	0.8841
Baseline+SDB	32.88	0.8896
Baseline+FDB+SDB	33.24	0.8970

## Data Availability

Not applicable.
